# Maximizing Performance and Stability of Organic Solar Cells at Low Driving Force for Charge Separation

**DOI:** 10.1002/advs.202305948

**Published:** 2023-12-01

**Authors:** Larry Lüer, Rong Wang, Chao Liu, Henry Dube, Thomas Heumüller, Jens Hauch, Christoph J. Brabec

**Affiliations:** ^1^ Institute of Materials for Electronics and Energy Technology (i‐MEET) Friedrich‐Alexander‐Universität Erlangen‐Nürnberg Martensstrasse 7 91058 Erlangen Germany; ^2^ Erlangen Graduate School in Advanced Optical Technologies (SAOT) Paul‐Gordan‐Straße 6 91052 Erlangen Germany; ^3^ Department Chemistry and Pharmacy Friedrich‐Alexander‐Universität Erlangen‐Nürnberg Nikolaus‐Fiebiger‐Straße 10 91058 Erlangen Germany; ^4^ Helmholtz‐Institute Erlangen‐Nürnberg (HI‐ERN) Immerwahrstraße 2 91058 Erlangen Germany

**Keywords:** electrostatics, exciton binding energy, high throughput methods, hybridization, open circuit voltage losses, organic photovoltaics

## Abstract

Thanks to the development of novel electron acceptor materials, the power conversion efficiencies (PCE) of organic photovoltaic (OPV) devices are now approaching 20%. Further improvement of PCE is complicated by the need for a driving force to split strongly bound excitons into free charges, causing voltage losses. This review discusses recent approaches to finding efficient OPV systems with minimal driving force, combining near unity quantum efficiency (maximum short circuit currents) with optimal energy efficiency (maximum open circuit voltages). The authors discuss apparently contradicting results on the amount of exciton binding in recent literature, and approaches to harmonize the findings. A comprehensive view is then presented on motifs providing a driving force for charge separation, namely hybridization at the donor:acceptor interface and polarization effects in the bulk, of which quadrupole moments (electrostatics) play a leading role. Apart from controlling the energies of the involved states, these motifs also control the dynamics of recombination processes, which are essential to avoid voltage and fill factor losses. Importantly, all motifs are shown to depend on both molecular structure and process conditions. The resulting high dimensional search space advocates for high throughput (HT) workflows. The final part of the review presents recent HT studies finding consolidated structure–property relationships in OPV films and devices from various deposition methods, from research to industrial upscaling.

## Graphical abstract

The introduction of non‐fullerene acceptors (NFA) into organic photovoltaics greatly reduces open circuit voltage losses Δ*V_OC_*. This review summarizes the current understanding of the origin of the driving forces for exciton (LE) dissociation and charge separation. Recent efforts are described to control and optimize the driving forces, with a goal of further reducing the voltage losses towards the radiative limit.

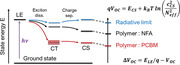

## 1 Introduction

Organic photovoltaic (OPV) systems present salient features such as light weight, flexibility, transparency, and color tunability, making them the ideal complement for PV market led by silicon technology, especially to address conflicting needs in land use for highly populated areas.^[^ ^1^, ^2^ ^]^ Recent years saw a dramatic boost of power conversion efficiencies (PCE) of OPV systems, now approaching 20%.^[^ ^3^ ^]^ This increase is mainly due to the introduction of non‐fullerene acceptors (NFA), providing complementary absorption with respect to the electron donor materials.^[^ ^4^, ^5^ ^]^ Free charges are thus not only formed by electron transfer from local excited states in the donor (LE_D_), but also by hole transfer from local excited states in the acceptor (LE_A_).^[^ ^6^ ^]^ This double role of the NFA phase as electron acceptor and light harvesting material led to a boost of the light absorption yield with a concomitant increase in short circuit currents (*J*_SC_) compared to previously deployed fullerene‐based acceptors with only weak absorption in the visible range.^[^ ^5^, ^7^ ^]^ Moreover, the skills of organic chemistry allow a fine‐tuning of frontier orbital energies such that both the driving force for exciton dissociation and the complementarity of light absorption can be optimized for each donor: acceptor pair.^[^ ^8^, ^9^, ^10^ ^]^ This has led to reduce the voltage losses Δ*V* = *qE*_*LE*,*x*_ − *V_OC_*, where *V_OC_* is the open circuit voltage, *E_LE,x_* is the lowest available optical bandgap (*x* ∈ {*D*, *A*}) and *q* is the elementary charge.^[^ ^11^ ^]^ Taking the improvements in *V_OC_* and *J_SC_* together, PCE comprising modern NFA systems has nearly doubled compared to the pre‐NFA era.

The question naturally arises whether OPV systems can be built with zero or even negative driving force for exciton dissociation.^[^ ^12^, ^13^, ^14^, ^15^ ^]^ Avoiding voltage losses caused by the need for exciton dissociation, such systems would parallel inorganic PV in terms of PCE, under the condition that high quantum yields for light absorption and exciton dissociation can be maintained.^[^ ^16^ ^]^ However, systematic variations of ionization potentials by chemical modification of donor and acceptor materials have so far shown that with driving forces approaching zero, there is a compromise between voltage losses and current losses.^[^ ^13^, ^17^, ^18^, ^19^ ^]^ The reduction of voltage losses upon reducing the driving force has been ascribed to the hybridization of interfacial charge transfer (CT) states with LE_A_ states increasing their electroluminescence quantum yield (ELQY).^[^ ^18^, ^20^, ^21^ ^]^ Based on the Energy Gap Law in the semiclassical Marcus picture of electron transfer, an intrinsic and unavoidable nonradiative voltage loss of ≈0.2 eV has been concluded for CT states of organic semiconductors.^[^ ^22^, ^23^ ^]^ However, it has also been noted that as driving forces decrease, the Boltzmann equilibrium between LE and CT states will increasingly shift toward the LE side. Thereby the ELQY of LE_A_ states, rather than CT states, becomes decisive for the voltage losses.^[^ ^19^ ^]^ Current losses at vanishing driving forces are explained by exciton dissociation rates approaching the intrinsic lifetimes of the LE_A_ states such that a minimum driving force of 0.2 eV has been found necessary to minimize current losses^[^ ^12^, ^17^, ^24^ ^]^

The energy of the interfacial charge transfer (CT) state is decisive for efficient exciton dissociation (LE→CT) and charge separation (CT→CS) at the same time. In D:A systems of high driving force, CT states and their associated energies are readily detected spectroscopically in EL and Fourier Transform Photocurrent Spectra (FTPS). However, as driving forces approach zero, the distinction of CT and LE states becomes difficult due to small energetic offsets and hybridization such that indirect methods have been deployed to assess the CT energy. Matching several experimental evidences (EL spectra, charge separation times, internal quantum efficiencies for charge extraction (IQE)) with a rate equation model, it has been shown that in a D:A blend with a low driving force (WF3:o‐IDTBR), charge separation is an energetic downhill process,^[^ ^12^ ^]^ which agrees with previous records for barrierless charge separation. In order to justify an energy gain upon charge separation despite the overcoming of Coulomb attraction, polarization effects have been invoked,^[^ ^25^ ^]^ which can be distinguished into higher exciton energies at a disordered interface,^[^ ^21^, ^24^, ^26^, ^27^ ^]^ band bending toward the D:A interface,^[^ ^28^ ^]^ and quadrupole moments caused by disorder at D:A interfaces comprising local dipolar NFA systems.^[^ ^13^ ^]^

In order to quantify the overall driving force for exciton dissociation, knowledge of the ionization energies and electron affinities (in a molecular picture also called the highest occupied and lowest unoccupied molecular orbital, HOMO and LUMO, energies, respectively) is needed. Two prominent approaches are available, namely photoelectron spectroscopy (PES) and cyclovoltammetry (CV).^[^ ^29^ ^]^ The former promotes electrons into a vacuum by hard UV radiation, while the latter induces a redox reaction in an electrolyte against a reference electrode. Photoelectrons emitted during PES do not carry information about geometric relaxation of the molecules, which can amount to 100 meV.^[^ ^30^ ^]^ Likewise, the presence of diffusion kinetics in the electrolyte of a CV experiment may be hard to interpret. Therefore, the experiment has been combined with spectroscopic detection of the redox products, because charged organic semiconductors present unique spectral features below the optical bandgap, allowing a background‐free detection.^[^ ^31^ ^]^

Operational stability of OPV systems is limited by photooxidation and morphological degradation. High CT energies,^[^ ^32^, ^33^, ^34^ ^]^ beneficial to avoid voltage losses, may sensitize singlet oxygen, one of the dominant causes for photooxidation on OPV.^[^ ^35^ ^]^ On the other hand, interfacial alignments for optimal quadrupole moments and optimal D:A gradients along the stack axis may not be the most thermodynamically stable molecular arrangements, entailing thermally induced performance loss during operation even in a well‐encapsulated state. The results presented so far identified the governing principles that limit PCE and operational stability. The question is thus: how can material science and device technology manage these principles such that the current limits can be overcome?

In this review, we present the state‐of‐the‐art and highlight promising approaches to go beyond. We first discuss the controversial role of exciton‐binding energy in the charge separation process. Then, we summarize recent findings on how polarization effects such as electrostatics influence the driving forces for both exciton dissociation (LE = >CT) and charge separation (CT = > CS). We discuss the role of hybridization of donor and acceptor states influencing the energies and radiative coupling of interfacial charge transfer states. Hybridization has also been shown to influence the energetic ordering of CT states in their singlet and triplet manifolds. We summarize promising approaches to deal with the “energy gap Law” in order to reduce nonradiative recombination of excitons being in thermal equilibrium with CT and CS states. We highlight that these phenomena depend on molecular structure but also on morphology, showing the importance of process conditions to optimize driving forces. Therefore, we present an overview of recent high‐throughput studies for various deposition methods, able to provide clear‐cut structure‐property relationships in a high dimensional experimental space.

## 2 Exciton Binding Energies and Driving Forces

### 2.1 Energy of the LE State: Exciton Binding Energy

There has been a long‐standing controversy about how Coulomb attraction can be overcome during exciton dissociation and charge separation in organic semiconductors. Excitons (spatially correlated electron‐hole pairs)^[^ ^36^ ^]^ in organic semiconductors are confined because the formation of an extended exciton wavefunction requires π–π overlap that is possible only in one or two dimensions due to the *C*_∞v_ symmetry of the basis state (p_z_ orbital). Even within the reduced dimensionality, further confinement of the exciton wavefunction is induced by steric hindrance or disorder, causing torsion along the π‐conjugated backbone that reduces the effective conjugation length.

In a neutral LE state (no net charge transfer), the average electron‐hole separation (“exciton size”)^[^ ^39^ ^]^ will be much smaller than the overall exciton wavefunction extension or persistence length.^[^ ^40^ ^]^ Therefore, substantial Coulomb binding between electron and hole will result (“exciton binding energy” *E_b_* ^[^ ^41^ ^]^), which must be overcome to form a charge‐separated state.^[^ ^42^, ^43^ ^]^

**Figure 1:**
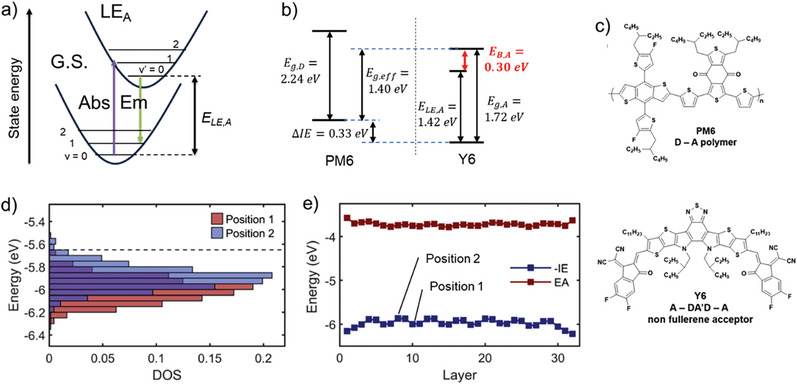
a) estimation of adiabatic transition energies of locally excited (excitonic) states and interfacial CT states, *E_LE_* and *E_CT_*, respectively, in the Frank–Condon picture by intersecting emission and absorption spectra (downward and upward arrows, respectively). For *E_LE_*, use photoluminescence and UV–vis data^[^ ^37^ ^]^; for *E_CT_*, use electroluminescence and Fourier Transform Photocurrent Spectroscopy‐ data. b) Evaluation of exciton binding energy *E_B,A_* of PM6:Y6 spin coated from CF:CN (chloroform + 0.5 v% chloronaphthalene) by comparing the optical bandgap *E_LE,A_* to the acceptor transport gap *E_g,A_*. Adapted with permission.^[^ ^31^ ^]^ Copyright 2022, Royal Society of Chemistry. c) Molecular structures of the donor PM6 and the acceptor Y6; d) Density of states (DOS) for holes (−IE) in a 10 nm thick model thin film based on molecular dynamics equilibration of the Y6 crystal structure, normalized to unit area. Dashed line indicates the DOS onset determined by photoelectron spectroscopy in air. e) Average ionization energy as a function of π‐stacking layer showing differential hole stabilization for the different lattice positions, which occur in an “…ABBA…” sequence. Adapted with permission.^[^ ^38^ ^]^ Copyright 2022, Springer Nature.

In **Figure** **1**a, E_LE,A_ is the available energy at time of exciton dissociation if thermalization (geometric relaxation of the initially formed state toward the new equilibrium geometry) and corresponding lattice polarization proceeds faster than exciton dissociation.^[^ ^30^ ^]^ In bulk heterojunction (BHJ) samples, this condition is not always fulfilled.^[^ ^44^ ^]^ In the presence of static disorder, exciton diffusion will be biased toward the low energy of the density of states (DOS) distribution^[^ ^36^, ^45^ ^]^ at the time of exciton dissociation; thus, the available energy will be overestimated by the method shown in Figure 1a.

Strong electron acceptors such as fullerenes can supply the necessary driving force by the formation of a charge transfer (CT) state with net charge transfer, justifying efficient exciton dissociation. However, the electron and hole remain in close vicinity even in the CT state, calling for a further explanation for charge separation of CT states into CS states. In D:A blends of high driving force involving fullerenes, delocalized or short‐lived high energetic CT states have been invoked^[^ ^46^ ^]^ in which Coulomb binding of the intermediate CT states would be below the thermal energy, justifying high charge separation yields.

The situation is different for low bandgap donor polymers and acceptors. Shifting the optical bandgap *E_LE,x_* of D:A blends more toward lower energies improves matching of the blend absorption with the solar spectrum. To accomplish this, the concept of intramolecular charge transfer (ICT) has been widely adopted, by which the conjugated backbone (for both donor and acceptor materials) is made of alternating donor and acceptor moieties.^[^ ^47^ ^]^ Excitons in such ICT systems are expected to show increased electron–hole separation, because the dwelling probability for electrons (holes) will be more on the acceptor(donor) moieties. One would therefore expect that in such systems, the exciton size is closer to the persistence length and consequently, *E_b_* is reduced compared to classical materials with non‐alternating units. Neusser et al.^[^ ^31^ ^]^ evaluated *E_b_* of the high‐performance NFA molecule Y6 (molecular structure shown in Figure 1c) by comparing the Y6 transport gap *E_g,A_* with *E_opt_* ≡ *E_LE_*:

(1) $$E_{b,A}=E_{g,A}\;-\;E_{LE,A}=IE_{A}\;-EA_{A}-E_{LE,A}$$

where *IE_A_* and *EA_A_* are the ionization energy and the electron affinity of the acceptor Y6 (structure shown in Figure 1c) in the solid state, respectively.^[^ ^31^ ^]^ In Figure 1a, we show how the exciton energy *E_LE,A_* is obtained in the Frank‐Condon picture. Measuring both *IE_A_* and *EA_A_* by spectroelectrochemistry, Neusser et al. are able to avoid ambiguities of standard cyclovoltammetry, because they can spectroscopically detect the number of oxidized/reduced species. Depending on preparation conditions for PM6:Y6 blends (molecular structure of PM6 shown in Figure 1c), they found *E*_*b*,*A*_ ≈ 0.3*eV*, suggesting that even for NFA materials with alternating D‐A moieties, a significant driving force for exciton dissociation is required. The authors noted that the value for the transport gap agreed well with the one observed from photoelectron spectroscopy.^[^ ^48^ ^]^ By analysing the blend PM6:Y6 (molecular structure of PM6 shown in Figure 1c), they found that the offset between the PM6 and Y6 ionization energies (IE_D_ and IE_A_, respectively) was ≈0.35 eV. As the energy difference of LE and CS states is given by:

(2) $$\Delta \;E_{CS,LE}=E_{LE}\;-\;E_{CS}=IE_{A}\;-IE_{D}-E_{b,A}$$

this finding yields near zero energy loss for charge separation in PM6:Y6. Explaining the high charge separation yield found in PM6:Y6 therefore seems to require contributions from entropy or from lower energetic tail states. If an exciton separates into two charged states, the number of possible arrangements of the charged states squares those of the single exciton, which can amount to a Gibbs free energy gain of 0.1–0.2 eV.^[^ ^26^, ^49^ ^]^ However, temperature‐dependent measurements did not confirm the entropy contribution^[^ ^9^ ^]^ for which reason the authors prefer the picture of sub‐bandgap tail states. Other authors have highlighted the importance of low interfacial disorder to achieve high charge separation yield in D:A systems of low driving force.^[^ ^50^ ^]^

Zhu and coworkers have calculated exciton binding energies by a quantum mechanics/embedded charge (QM/EC) method.^[^ ^24^ ^]^ According to these authors, polarization effects of ionic (charged) states in the solid state can overcompensate Coulomb attraction in the exciton, which can even lead to negative exciton binding energies. According to the QM/EC calculations, the amount of polarization depends on the amount of π–π overlap. The crystal structure of the well‐known NFA molecule Y6 (for molecular structure see Supporting Information) shows strong π–π overlap of terminal as well as central moieties, whereas the NFA molecules ITIC and IT‐4F show only terminal overlap^[^ ^51^, ^52^ ^]^ Consequently, *E_b_* values ≈0.2 to 0.4 eV are calculated for ITIC and IT‐4F, whereas a negative exciton binding energy of about −0.12 eV is found for Y6. The authors support their calculations by temperature‐dependent photoluminescence spectroscopy, finding an increase of PL intensity for ITIC at low temperatures while the PL intensity of Y6 decreases. Furthermore, they build an OPV device comprising only Y6 as active layer and obtain a PCE of 0.42%, orders of magnitude higher than the corresponding ITIC only based device. This demonstrates intrinsic charge separation in the pure Y6 phase without the need of additional driving force from a donor with a higher HOMO level.^[^ ^13^ ^]^

Price and co‐workers have confirmed intrinsic charge separation in pure Y6 and performed quantum chemical simulations.^[^ ^13^, ^38^ ^]^ According to combined quantum mechanical/molecular dynamic simulations, the driving force for free charge generation comes from the presence of two phases with slightly different ionization energies. Figure 1d shows that on average, a driving force for hole transfer of ≈100 meV results. Due to the “ABBA”‐type stacking in the lattice, see Figure 1e, this can explain intrinsic charge separation in the Y6 phase.

In summary, the best currently available methods for evaluating the transport gap (spectroelectrochemistry and ambient photoelectron spectroscopy) agree that even modern NFA materials with alternating D‐A moieties, still show significant exciton binding energies that need to be overcome during charge separation. On the other hand, quantum chemical calculations and experimental charge separation yields would rather point to near–zero exciton binding energies. Both entropy and disorder contributions have been suggested to unify the pictures, but again ruled out by other studies. Searching in Equation (1) for possible artifacts, it would be a surprise if the transport gaps, obtained from two entirely different methods, would yield the same systematic offset. Likewise, the estimation of *E_opt_* is done in the Frank–Condon picture in the limit of displaced, undistorted oscillators, which is justified for organic semiconductors. We therefore conjecture that more insight be obtained with respect to entropic contributions and the role of disorder, possibly in a high throughput fashion.

### 2.2 Energy of the CT State: Polarization and Electrostatic Roll‐Off

**Figure 2:**
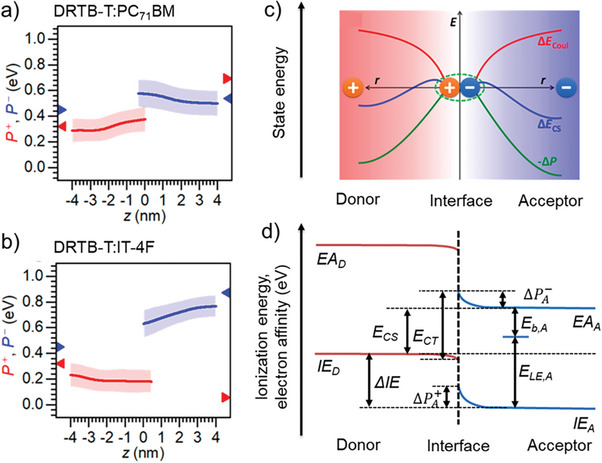
a) Evolution of electrical polarization for electrons in the acceptor phase, and for holes in the donor phase (blue and red curves, respectively), in DRTB:PC71BM, according to molecular dynamics (MD) simulations. Shadows refer to the confidence intevals for polarization curves obtained at different MD snapshots. Redrawn with permission after ref. [25] b) same as a), for DRTB‐T:PC71BM c) Electrical polarization (ΔP, green curve) can counteract Coulomb attraction of interfacial CT state (Δ*E_coul_*), red curve, leading to barrierless charge separation (Δ*E_CS_*), blue curve. Adapted with permission.^[^ ^25^ ^]^ Copyright 2020, American Chemical Society. d) Energies of states relevant for the overall driving force for exciton dissociation in donor‐acceptor blends.^[^ ^48^, ^53^ ^]^ IE and EA are ionization energies and electron affinities, respectively; *E_CS_* is the energy of the fully charge separated state. Subscripts A and D refer to acceptor and donor, respectively, while superscripts e and h refer to electrons and holes, respectively. B means interfacial band bending due to bulk stabilization by quadrupole moments. *E_B_* is the exciton binding energy in the solid‐state balancing Coulomb attraction and lattice polarization. Solid blue lines: IE and EA of acceptor adopting a morphology leading to quadrupole moments.

Interfacial CT states show near unity charge transfer across the D:A interface. However, there is still substantial Coulomb binding in the nearest neighbor CT complexes that must be overcome for complete charge separation. The observation of fast charge separation kinetics^[^ ^12^ ^]^ and barrierless charge separation^[^ ^13^ ^]^ seems to suggest the presence of one or more motifs able to counterbalance or even overcome the Coulomb interaction in CT states. When discussing exciton binding energies in the previous chapter, we highlighted polarization effects in the solid state that can counteract Coulomb binding. According to the theoretical studies by Zhu and co‐workers,^[^ ^24^ ^]^ the amount of polarization is related to the amount of π–π interaction. Polarization effects should therefore be maximized in the ordered bulk, whereas they should be reduced in regions close to the interface. The effect is rendered by the QC/QMM calculations shown in **Figure** **2**c, where the ionization energies are increased toward layer numbers 1 and 32. Reduced polarization at the D:A interface therefore acts as a driving force toward charge separation into the more strongly polarizable bulk.

**Figure 3:**
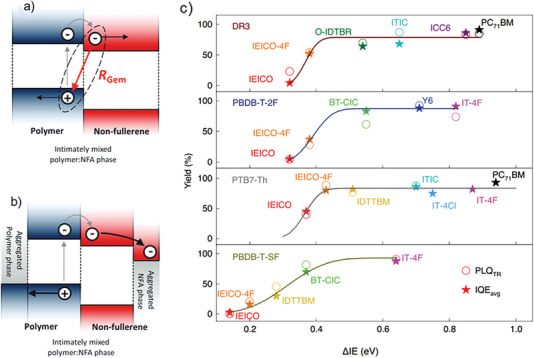
a) Intimately mixed polymer: NFA phase cannot surmount Coulomb binding energy of interfacial CT states, causing geminate recombination (*R*_gem_). b) Bulk stabilization of aggregated phase provides additional driving force for charge separation, reducing geminate recombination. Reproduced with permission.^[^ ^28^ ^]^ Copyright 2019, Wiley VCH. c) Dependence of internal quantum efficiency (IQE) on ΔIE for different D:A combinations. Reproduced with permission.^[^ ^48^ ^]^ Copyright 2021, Springer Nature Ltd. These authors assume zero *E*_b_, therefore they can use the fitting curves (solid lines) to determine the amount of quadrupole‐induced band bending for the different donor polymers.

Polarization effects have been modeled by Tu et al. for a series of donor:acceptor combinations by means of molecular dynamics simulations.^[^ ^25^ ^]^ For the system DRTB‐T:PC_71_BM, they found a small and destabilizing effect of polarization when going from the interface to the bulk, see Figure 2a; in contrast, for the NFA system DRTB‐T:IT‐4F, they found a strong and stabilizing effect, see Figure 2b. In Figure 2c, it is shown that a strong and stabilizing polarization effect in the bulk can exceed Coulomb attraction, leading to an overall positive driving force when going from the CT state to the CS state and thus to barrierless charge separation. Figure 2d summarizes the energies contributing to exciton binding, exciton dissociation, and charge separation. As shown by Cha and co‐workers,^[^ ^28^ ^]^ barrierless charge separation from a CT state becomes possible if an intermixed phase is formed between relatively pure donor and acceptor phases. This is due to the lack of bulk stabilization for both donor and acceptor, which results in an increase in the energy of the interfacial CT state relative to that of the CS state, see **Figure** **3**a,b. Moreover, Karuthedath and co‐workers.^[^ ^48^ ^]^ have considered intramolecular charge transfer (ICT) in modern NFA systems built from A‐D‐A moieties.^[^ ^48^ ^]^ In the presence of interfacial disorder, the resulting local dipole moments can form quadrupole moments, which belong to the electrostatic contributions to polarization predicted in Figure 2. The electrostatic contribution from quadrupole moments is however reduced at the D:A interface, such that quadrupole moments present another strong motif to drive charge carriers away from the interface and into the bulk. Karuthedath et al analyzed a large number of D:A blends and found typical quadrupole induced driving forces toward the bulk in the range of 0.3–0.4 eV, see Figure 3c.

**Figure 4:**
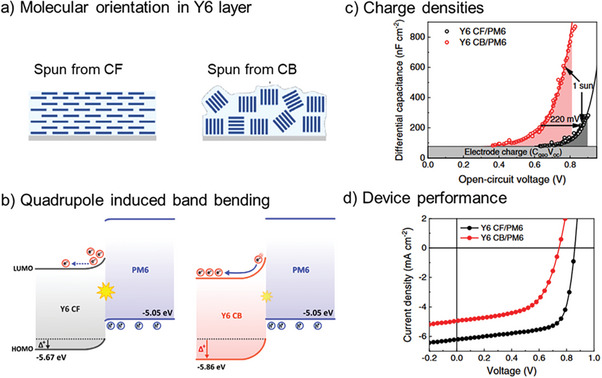
Morphology‐dependent quadrupole interactions in PM6:Y6 bilayer devices a) Sketch of molecular orientations obtained from different solvents b). Frontier orbital level bending due to different amounts of quadrupolar interaction in the Y6 phase of PM6:Y6 bilayer film spun from CF and CB (left and right, respectively). c) Differential capacitance measurements of devices comprising the active layers from panel (a). d) Corresponding current–voltage curves. Reproduced with permission.^[^ ^53^ ^]^ Copyright 2023, Springer Nature Ltd.

In a recent study, Fu and co‐workers looked more closely at the PM6:Y6 blend and demonstrated that the effect of quadrupolar moments on electrostatic stabilization depends drastically on molecular orientation.^[^ ^53^ ^]^ They prepared films and devices from PM6:Y6 in a bilayer architecture, using a film transfer technique (see final chapter of this review). This allowed them to specifically change the morphology of the acceptor but not of the donor. Preparing Y6 films from CF solutions, they obtain small crystallites and a preferential face‐on orientation, while in films prepared from chlorobenzene (CB), a high boiling point solvent, they obtained larger crystallites with a more random orientation. Sketches of the nanomorphology, as obtained from grazing incidence wide angle X‐ray scattering (GIWAXS), are shown in **Figure** **4**a. Using ambient photoelectron spectroscopy and Kelvin probe, they showed that in Y6 films prepared from CB, both *IE_A_* and the Fermi energy are shifted downward by ≈0.2 eV compared to films prepared from CF. As changes in *E_opt_* amount only to 0.03 eV, the authors conjecture that also *EA_A_* is shifted downward by ≈0.2 eV. These parallel shifts of both *IE_A_* and *EA_A_* point to an electrostatic effect. Combining DFT and molecular dynamics (MD) simulations, they showed that quadrupole moments cause stronger electrostatic effects in large crystallites with more random orientation than in small face‐on oriented crystals, see Figure 4b, justifying the experimental observations.

By measuring differential capacitance and subtracting the expected electrode charge, they found that under the same illumination conditions, PM6:Y6 prepared from CB was able to store a much higher charge density in the active layer than that prepared from CF, see Figure 4c. This points to strongly reduced recombination in PM6:Y6 prepared from CF. In agreement with the picture developed by Karuthedath et al, Fu and co‐workers explained the reduced recombination by electrostatic repulsion from the D:A interface because of a reduced contribution of quadrupolar effects at the D:A interface compared to the bulk, see Figure 4b. The reduced recombination can also be seen from the fact that in Y6 CB:PM6, the open circuit voltage (*V_OC_*) is only reduced by ≈120 mV, see Figure 4d, although the effective gap, due to increased quadrupolar interaction, is reduced by 200 meV.

In summary, there is now a growing body of evidence that in high performance OPV blends, various polarization effects provide driving forces for electron–hole separation away from the D:A interface toward the bulk.^[^ ^25^ ^]^ These driving forces are often strong enough to counterbalance or even exceed the Coulomb binding energy in interfacial CT states, justifying barrierless charge separation and reducing recombination. A complication arises from the fact that it is difficult to predict quadrupolar moments from first principles, as they depend on both material combination and process conditions.

### 2.3 Energy of the CS State: Balancing Electrostatics

A CS state is described by a fully relaxed donor cation and a fully relaxed acceptor anion at infinite distance of each other such that Coulomb interaction between them is fully screened.^[^ ^54^ ^]^ In the previous section, we have shown that quadrupolar moments can provide a motif for separation of CT into CS states. The price we pay is a decreased energy of the CS state:

(3) $$E_{CS}=E_{g,eff}\;=IE_{D}\;-EA_{A}$$

As *E_CS_* is equivalent to the effective gap *E*_*g*,*eff*_, quadrupole moments will reduce the maximum achievable *V_OC_*. This factor is usually not considered when optimizing the performance of a given blend by varying the process conditions. Although the electrostatic roll‐off at the interface may lead to reduced recombination and higher QFLS, the higher QFLS may not translate into a higher *V_OC_*. Considering only the trade‐off between interfacial recombination and effective gap, the optimal D:A combination in an OPV active layer may be obtained by using materials whose vacuum IE have zero offset, that is, that would provide a driving force for charge separation only by virtue of quadrupolar moments. However, we also need to consider the fact that at the D:A interface, the quadrupolar moments are reduced but not to zero, thus influencing the energy of the interfacial CT states. Hence, quadrupolar moments lead to *E_CT_* and *E_CS_* becoming intimately entangled. This complicates performance optimization, as it has been shown that for given LE and CT lifetimes, there are optimum driving forces from LE→CT and from CT → CS.^[^ ^19^ ^]^

Apart from the D:A interface, also the interface between the active layer and the extraction layers requires attention. An optimal quadrupole‐driven acceptor would show electrostatic roll‐off toward the hole transporting layer (HTL), while it would show flat conditions toward the electron transporting layer (ETL). It would be experimentally very tedious to provide such a material satisfying all those conditions by means of chemical synthesis because we lack predictive capacity for nanostructure driven by van der Waals interaction. An alternative may thus be to regulate the formation of favourable morphologies that are close to the HTL and ETL layers by controlling process conditions.

In summary, providing the optimal driving forces for exciton dissociation (LE → CT) and final charge separation (CT → CS) requires a careful adjustment of both chemical structure and process conditions. The resulting high dimensional search space advocates for high throughput (HT) workloads. With respect to chemical structure, More and co‐workers^[^ ^55^ ^]^ have presented an artificial intelligence (AI) driven method to improve the predictive capacity of DFT calculations of frontier levels (equivalent to IP and EA in vacuum). Usually, AI methods require a large amount of training data, which the authors tackle by a method called “transfer learning”: they train a first convolutional neural network (CNN) on the Harvard Clean Energy Project (HCEP, 500 000 molecules) to predict the DFT‐calculated frontier orbital levels from SMILES (simplified molecular input line entry specification) representations of chemical structure. Then, they fine‐tuned the model by training on 194 experimental fronter orbital energies. They obtained an uncertainty below 200 meV, which outperforms direct predictions by DFT. Although one would wish to achieve an accuracy on the order of k_B_T (≈25 meV at room temperature), the method has demonstrated its potential. Its accuracy will be improved once a larger and more consolidated experimental dataset is available.

With respect to process conditions, the complication arises from the need to measure the frontier orbital levels as a function of process conditions, which contradicts a HT workflow. One way to tackle the problem is to match experimental evidence measured in the actual device to model simulations in which the state energies are model parameters. Gasperini et al have used a rate equation model involving LE, CT, and CS states to predict available experimental evidence in WF3:o‐IDTBR devices, namely the EL spectra, the IQE at a given excitation wavelength and the charge separation time, found by the transient Stark effect.^[^ ^12^, ^26^ ^]^ It was found that the model was only able to match the available evidence if the CT energy was assumed higher than the CS energy, which means barrierless charge separation. Azzouzi et al. have extended this method by integrating it with a 1D drift‐diffusion model, which allows them to model electrical device performance, including the fill factor (FF).^[^ ^56^ ^]^ These authors have demonstrated their method by using a limited set of material combinations. Using machine learning algorithms, these methods can be generalized. Digital twin approaches add value to experimental evidence by reproducing them with appropriate physics models, providing end‐to‐end uncertainty quantifications for the crucial model parameters. The model parameters are optimized as new evidence is added by machine learning methods such as reinforcement learning. Therefore, digital twin approaches may prove helpful in finding crucial but hard to access material parameters such as the exciton binding energy and the available driving force for charge separation.^[^ ^57^ ^]^

## 3 Hybridization and Dynamic Equilibration

Due to the momentum conservation rule, pure charge transfer transitions carry no oscillator strength; interfacial CT states can therefore not be resonantly excited if their creation would result in a full charge transfer. However, quantum mechanics requires that if two electronic systems are in sufficiently close proximity, then their frontier orbitals will split in energy, creating hybrid orbitals with mixed (hybrid) properties of both molecules. If hybridization occurs between a donor and an acceptor molecule at the D:A interface, then the HOMO of the combined (D:A) electronic system, dominated by the donor HOMO, will also attain weak contributions from the acceptor HOMO.^[^ ^18^, ^58^ ^]^ As a consequence, there will be nonzero charge transfer even in the electronic ground state of the D:A system.^[^ ^59^, ^60^ ^]^ Likewise, the LUMO of the D:A system, dominated by the acceptor LUMO, will weakly attain contributions from the donor LUMO as well, such that an electron in this LUMO will not exclusively reside on the acceptor. Thus, even if an electron is promoted from the D:A HOMO to the LUMO, charge transfer will not be complete, which results in a nonzero oscillator strength, making a resonant excitation of CT states weakly allowed. Hybridization of D and A HOMO and LUMO orbitals is thus the reason why CT states can be observed in FTPS and, via the Strickler and Berg relation between induced absorption and spontaneous emission, also in EL spectra.^[^ ^61^ ^]^

**Figure 5:**
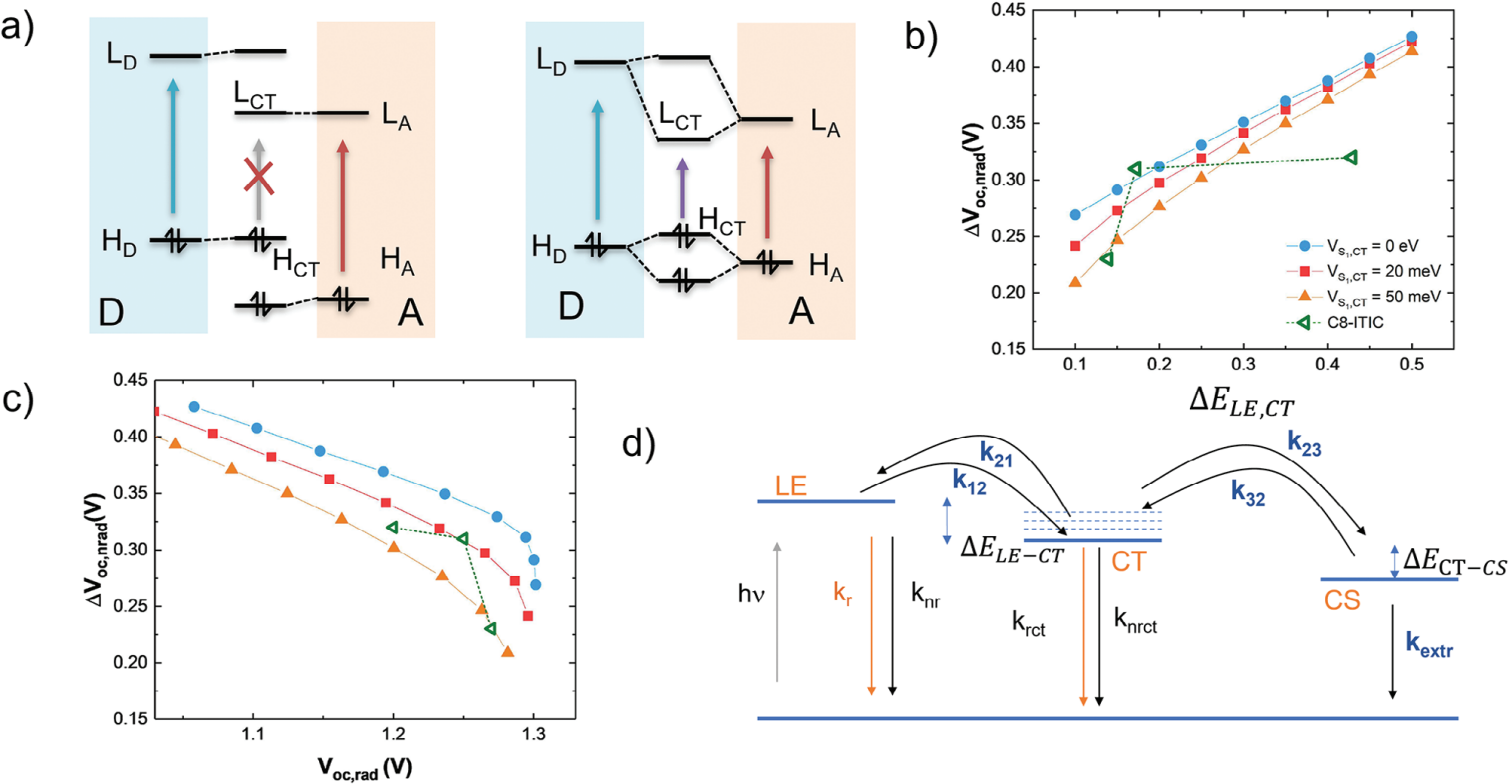
a) Hybridization of Frontier orbitals at the D:A interface in a one‐electron picture. Left: large energy offset–weak hybridization – forbidden transition. Right: small energy offset–strong hybridization–allowed transition. b) and c) evolution of the nonradiative voltage losses and the radiative *V*_OC_, respectively, with the energetic offset between LE and CT state Δ*E*_*LE*,*CT*_ and the amount of hybridization V_S,CT_. B,C) Reproduced with permission.^[^ ^18^ ^]^ Copyright 2019, American Chemical Society. d) Three‐states representation to predict the quasi‐Fermi level splitting from the energy levels, their degeneracy, and the recombination pathways to the ground state.

In a simple picture, the splitting of frontier orbital levels can be explained by the concept of linear combination of atomic orbitals to molecular orbitals (LCAO‐MO): linear combinations of opposite signs will have strongly different energies if the number of nodal plains is the same. As similar number of nodal plains implies similar energies, it is expected that the amount of hybridization increases upon reducing the energy difference in the donor and acceptor frontier orbitals. This is shown in **Figure** **5**a: on the left, *H_A_* and *H_D_* have different energies and weak hybridization, while on the right, both *H_A_* and *H_D_* are similar leading to strong hybridization. In this picture, it is expected that D:A systems with small driving force have interfacial CT states showing stronger radiative coupling to the ground state. According to Rau's formula derived from detailed balance conditions,

(4) $$\Delta \;V_{OC,nr}=\;-k_{B}T\cdot ln\left(ELQY_{ext}\right)$$

nonradiative voltage losses are linked to the external ELQY; brighter CT states should therefore reduce nonradiative voltage losses.^[^ ^61^ ^]^ Using time‐dependent density functional theory, Han et al. have shown that in DRTB‐T:IT‐4F, a low driving force blend, the interfacial CT states can borrow considerable oscillator strengths from the local excitation (LE) state.^[^ ^20^ ^]^

**Figure 6:**
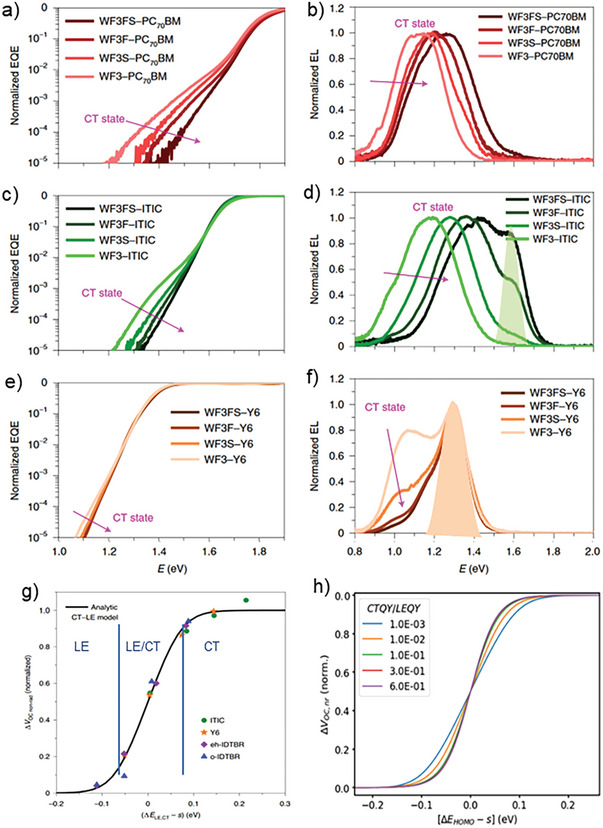
a–f) FTPS‐EQE (left) and EL (right) spectra of D:A blends with three different acceptors (rows) combined with different donor polymers, where color depth of the curves increases for decreasing donor HOMO energy. g) evolution of the normalized nonradiative voltage loss of the samples from Figure 3 with the standardized HOMO offset (symbols) and prediction from an analytical two states model (black line). Vertical blue lines indicate approximate borders for the three regimes; for each regime the main contribution to ELQY is indicated. h) Dependence of the normalized nonradiative voltage loss on the ratio of the luminescent quantum efficiencies of the CT and LE state (CTQY and LEQY, respectively). For a ratio > 0.1, a unique curve is produced. Reproduced with permission.^[^ ^19^ ^]^ Copyright 2020, Springer Nature Ltd.

According to Boltzmann statistics, a dynamic equilibrium between CT and LE states will be observed if their energy is close to the thermal energy *k_B_T*, where *k_B_* is the Boltzmann constant, and *T* is the absolute temperature. Classen et al.^[^ ^19^ ^]^ studied a series of different NFA molecules combined with a series of four WF3 donor polymers derivatives, in which the variation of chemical substitutions allowed to shift the HOMO levels in steps of ≈60 meV, keeping morphological changes at a minimum. EL spectra of most D:A combinations are shown in **Figure** **6**a–f. They clearly exhibit two distinct contributions, namely a sharp band at the same energy as PL from the pure low bandgap (acceptor in this case) material (indicated by shaded areas in Figure 6d,f, and a broad, red‐shifted component whose energy strongly depends on the donor. It follows that under charge injection conditions, LE_A_ states are populated, a clear sign of thermal equilibration. The population ratio of CT and LE states ($\frac{p_{CT}}{p_{LE}}$), following the Boltzmann distribution, is given by:

(5) $$K\;=\frac{p_{CT}}{p_{LE}}\;=g_{21}\;\cdot exp\left(\Delta E_{LE,CT}/k_{B}T\right)$$

where *k_r,LE_* and *k_r,CT_* are the rate constants for radiative emission from LE and CT states, respectively, and *g_21_* is the ratio of the degeneracies of LE and CT states. The degeneracies of LE and CT states refer to the available number of LE and CT states that are in a detailed balance relationship. In highly performant bi‐continuous networks of donor and acceptor domains (so‐called bulk heterojunctions, BHJ), we can assume that the exciton diffusion lengths exceed typical domain sizes (otherwise IQE values of high driving force D:A systems could not approach unity).^[^ ^62^, ^63^ ^]^ Detailed balance can thus be installed across the whole bulk. Under this assumption, we can identify the degeneracy ratio *g_21_* with the ratio between interfacial states and bulk states, governed by geometrical factors such as the average domain size and their shape (ellipticity). Hence, although CT states may have a lower energy than LE states, the Boltzmann equilibrium may still be on the LE side due to the degeneracy ratio *g_21_*.

Classen et al. have used a homogeneous rate equation model involving only LE and CT states and their respective deactivation pathways to predict nonradiative voltage losses as a function of the driving force for exciton dissociation.^[^ ^19^ ^]^ They found that all studied systems can be described by three regimes, shown as blue vertical lines in Figure 6g: for high driving forces, a soft dependence of *ΔV_nr_* of ≈0.2 V eV^−1^ is found, in line with predictions from the Energy gap Law in CT states. For intermediate driving forces, sigmoidal behavior is found where both LE and CT states contribute to *ΔV_nr_*. For all studied acceptors, a maximum slope of ≈0.8 V eV^−1^ is observed at the fitted inflection point, in line with predictions from the two‐level model at room temperature. For vanishing driving forces, a lower limit of *ΔV_nr_* was observed, given by the PLQY of the LE_A_ states. It was found that most acceptors exhibit low IQE values in this regime, except the acceptor Y6, exhibiting the longest exciton lifetimes of all NFA molecules studied. It was concluded that in the regime of low driving forces, the luminescent properties of the interfacial CT state become less relevant, as due to the higher degeneracy of LE compared to CT states, most recombination occurs through LE states. To further reduce voltage losses, it is therefore of utmost importance to encounter NFA systems with long exciton lifetimes, that is, with low rates for nonradiative recombination.

Another crucial aspect of hybridization between LE and CT states has been highlighted by Gillett and co‐workers.^[^ ^64^ ^]^ Under open circuit conditions, free charges can only disappear through reformation of CT states. If electrons and holes originate from different photogeneration events (so‐called non‐geminate charge pairs), their unpaired spins will be uncorrelated, leading to a population of ≈75% of ^3^CT states in the triplet state, and ≈25% of ^1^CT states in the singlet state, following spin statistics. In D:A pairs with low driving forces, this may be a problem, because the energy of the CT states–of either spin multiplicity–may be above that of the NFA triplet states such that triplet formation may present an irreversible loss channel. Gillett and co‐workers performed quantum chemical simulations of D:A dimer and calculated the energy of the ^3^CT and ^1^CT states. In the D:A blend PTB7‐Th: IEICO‐2F, they found an inversion of the energetic ordering, with ^3^CT attaining a higher energy than ^1^CT. According to their calculations, this energetic inversion was caused by hybridization of LE and CT states, which also reduced the coupling of the ^3^CT state with the molecular triplet of the acceptor. In this case, the ^3^CT state has time to re‐dissociate into free carriers, which was demonstrated by femtosecond transient absorption spectroscopy. In contrast, in PM6:Y6 blends, no such energetic inversion of ^3^CT and ^1^CT was found.^[^ ^13^, ^64^, ^65^ ^]^

## 4 Strategies for Overcoming the Energy Gap Law for LE States

The energy gap Law states that the rate of nonradiative decay increases exponentially with the energy gap between excited state and ground state.^[^ ^61^ ^]^ Given that the ELQY is defined as:

(6) $$ELQY=k_{r}/\left(k_{r}+k_{nr}\right)$$

and that the ELQY controls Δ*V_nr_* according to Rau's formula (Equation 2), the energy gap Law constitutes a formidable barrier to reducing the nonradiative voltage losses in D:A systems with low driving forces. A detailed discussion of the rate of nonradiative recombination in CT states has been given by Benduhn and co‐workers.^[^ ^66^ ^]^ However, for D:A systems with low driving forces, the excited state population will reside on the LE rather than the CT states,^[^ ^67^ ^]^ warranting a specific discussion on how to control the nonradiative recombination rate in LE states.

**Figure 7:**
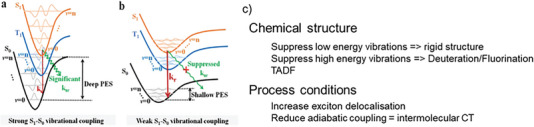
Overcoming the limitations imposed by the Energy Gap law. a) Coupling of the electronic transition with high energy vibrations leads to steep potential energy surfaces (PES) with strong nonradiative coupling. b) reduced coupling to high energy vibrations leads to flat PES with weaker nonradiative coupling. Reproduced with permission.^[^ ^69^ ^]^ Copyright 2021, Wiley‐VCH. c) summary of strategies to overcome the energy gap law that can be actuated by either chemical structure or process conditions. References for the mentioned principles are given in brackets.

The Energy gap Law, for a given energy difference, depends on structural factors, influencing the vibronic coupling and the anharmonic coupling among available vibrations in the system.^[^ ^68^ ^]^ As shown in **Figure** **7**c, both chemical structures and process conditions provide ways to reduce *k*_nr_ for a given energy difference of the electronic states, encouraging intense research efforts toward overcoming the limitations given by the Energy gap Law.^[^ ^56^, ^69^ ^]^

Following Born‐Oppenheimer's rule, internal conversion proceeds isoenergetically, converting an S_1_ state in its lowest vibronic state into a highly vibronically excited S_0_ state, see Figure 7a ^.[^ ^70^ ^]^ In the absence of anharmonic coupling, IC would be reversible because the available potential energy could not be redistributed among the available vibrational degrees of freedom. Therefore, the availability of vibrational quanta that are anharmonically coupled to the dominant vibronic modes, is the condition for nonradiative recombination.^[^ ^36^ ^]^

Low energy vibrations are typically induced by torsional motion about C–C single and double bonds. Given the sinusoidal shape of the potential energy along the torsion angle axis^[^ ^71^ ^]^ strong anharmonicity can be expected. Therefore, suppression of torsional motion is supposed to reduce nonradiative recombination. In fact, most high performance NFA systems such as Y6 inhibit torsional motion in large portions of their conjugated backbones. High energy vibrations are even more detrimental than low energy ones, because a single quantum with an energy exceeding the thermal energy can cause a large irreversible energy loss.^[^ ^12^ ^]^ In organic conjugated materials, the highest energy vibrations are observed in C─H bonds. Therefore, perfluorination and deuteration have been used to slow down the vibrational frequencies, reducing these vibrational energies. Partial fluorination is known to have a beneficial effect on controlled growth of a desired microstructure.^[^ ^72^ ^]^ It is however not sufficient for a strong reduction of *k_nr_*, and deuteration is expensive and therefore it is not suitable for photovoltaic materials aimed for large area applications. Another important high energy vibration is the C─C stretch vibration at ≈0.18–0.24 eV for aromatic systems, caused by a change of bond length alternation (BLA) when going from the ground to the excited state.

Furthermore, thermally activated delayed fluorescence (TADF) due to re‐formation of singlet states from triplet states has been used to reduce the amount of nonradiative recombination.^[^ ^57^ ^]^ However, the large reorganization energies associated with the TADF process discourage the use of this feature for photovoltaics. Apart from using the triplet pathway, long excited state lifetimes can also be provided by molecular moieties that in polar environments lead to a stabilization of the excited state by twisted intramolecular charge transfer (TICT). The phenomenon has been recognized and thoroughly studied first in p‐dimethylaminobenzonitrile but has also been extended to many different chromlophores, for example, lately to hemi‐thioindigo.^[^ ^73^, ^74^ ^]^ It has been found that the twisting efficiently decouples the excited state from the ground state, guaranteeing long lifetimes which makes it a promising system for photoswitching but also for photovoltaic applications. A further approach has been shown by Zhang and co‐workers, using multiple boron (B)‐and nitrogen (N)‐atoms embedded polycyclic heteroaromatics featuring hybridized p‐bonding/ non‐bonding molecular orbitals. Exploiting 2D conjugation, these moieties induce a multi‐resonance effect on the peripheral skeleton for the non‐bonding orbitals, creating shallow potential energy surfaces to eliminate the high‐frequency vibrational quenching, see Figure 7b.^[^ ^56^, ^69^ ^]^

Apart from structural details of single molecules, supramolecular arrangements also can help reduce nonradiative recombination. Increasing exciton delocalization^[^ ^18^, ^61^ ^]^ by forming strong J aggregates will reduce the coupling to vibrational degrees of freedom and at the same time lead to stronger radiative coupling at lower energy absorption and emission. This is particularly the case for NFA molecules like Y6. In contrast, conjugated polymers are typically described by weak H aggregation, where the excitonic splitting is less than the dominant vibrational frequency. Finally, Xue and co‐workers have described an approach to suppress the nonadiabatic coupling by formation of intermolecular charge transfer aggregates.^[^ ^18^ ^]^ As in the case of TADF, the amount of reorganization must be kept under control to avoid exciton trapping.

In summary, we have mentioned various strategies to overcome the limitations of the Energy gap Law for nonradiative recombination^[^ ^16^, ^75^, ^76^ ^]^ Some of these strategies are already actively being pursued in NFA materials, such as rigidifying the molecular backbone and forming strong J aggregates, others are only beginning to be explored, such as forming dual resonances in 2D conjugation. However, most of these approaches have side effects for practical applications, most probably a combination of these factors will have to be fine‐tuned for maximum performance. Moreover, multi‐excitonic systems may be incorporated to cover the low energy part of the solar spectrum, such that higher optical bandgaps may be used for the OPV systems.^[^ ^77^, ^78^, ^79^ ^]^ As both chemical structure and process conditions are concerned, a huge experimental space will result, which calls for high throughput methods combined with machine learning approaches, enabling inverse molecular design to predict the optimal molecule and corresponding process conditions.^[^ ^80^, ^81^ ^]^

## 5 High Throughput Workflows to Assess Processing‐Morphology‐Performance Nexus

**Figure 8:**
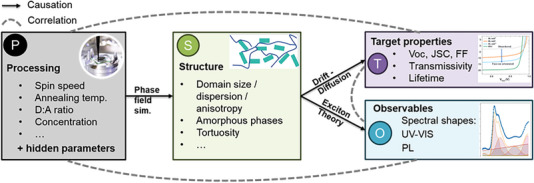
Causal pathways between processing conditions (P), structural features (S), target properties (T) and observations (O) (black arrows). Any pathway along a causal chain will yield a correlation (gray dashed lines) that can be quantified by high throughput studies. The causal pathways can also be predicted by approximate physical models (given along the arrows).

Figure 6 shows that if driving forces are small, then dynamic equilibria are formed between LE, CT, and CS states, following Boltzmann statistics under consideration of the degeneracy ratio between interfacial and bulk states. Under a *V*_OC_ condition (no extraction field), this means that the quasi‐Fermi level splitting (QFLS), and thus *V*_OC_, is limited by the recombination rates of both LE and CT states.^[^ ^56^, ^70^ ^]^ However, for maximum photovoltaic performance, not only *V*_OC_ but also *J*_SC_ must be maximized, and must be robust against operational degradation. Maximum *J*_SC_ requires maximum absorption yields and minimum yields of geminate recombination, while for maximum FF, charge extraction must outperform recombination even at moderate extraction fields, which points to the importance of the extraction mobility along the stack direction. All the above factors are not intrinsic material properties that could be measured once & for all for a given material, but they critically depend on the nanomorphology of the active layer,^[^ ^82^, ^83^ ^]^ which often shows a dramatic dependence on the preparation conditions.^[^ ^80^, ^84^, ^85^ ^]^ As shown in **Figure** **8**, critical morphological features are the domain size, the domain anisotropy, and the extension as well as relative amount of amorphous regions in both donor and acceptor phases. The community has recognized the importance of nanomorphology to understand photovoltaic performance by utilizing diffraction methods such as GIWAXS, which is state of the art in characterization of emerging PV materials.^[^ ^86^ ^]^

Figure 8 details the expected causal relationships between processing, structure, and performance.^[^ ^57^ ^]^ Processing conditions determine the formation of structural features such as the domain size, their anisotropy and orientation, their purity, and the fractal dimension of the D:A interface. The ensemble of structural features determines crucial photophysical parameters such as the state energies and their coupling, which in turn yields generation and recombination rates of LE, CT, and CS states, and the microscopic and macroscopic charge carrier mobility. These parameters finally determine the shape of the current‐voltage curve. Although in the large ensemble limit (valid for large area PV devices), these relationships are deterministic, substantial scattering of device performance data is often observed even following the same recipe in device formulation. This is due to the fact that some “hidden” process parameters with strong influence on the structural features are not under control, as indicated in Figure 8.

Discovering the dependence of structure on processing conditions, and of performance on structure, is experimentally very tedious due to the high dimensionality of the experimental space. For this reason, high throughput workflows have been introduced and combined with Bayesian optimization in order to minimize the number of experiments necessary to encounter an optimum. Figure 8 shows that such workflows probe the correlation along a causal or non‐causal pathway, even though the causal chain itself remains a black box. In order to understand detailed causal relationships, morphology–sensitive probes must be present during high throughput optimization. However, the above‐mentioned diffraction methods are difficult to be implemented into an autonomously operating robotic line as both, the data collection and evaluation, require expert intervention.

**Figure 9:**
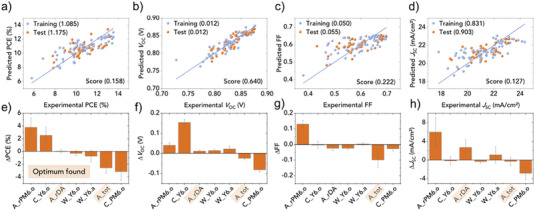
Gaussian Process Regression (GPR) to predict PCE, *V*_OC_, FF, and *J*_SC_ of PM6:Y6 solar cells from shapes of the UV–VIS spectra of the active layer (a–d, respectively). Root mean square errors (RMSE) for the test and training datasets are given in brackets. The R2 score of a five‐fold crossvalidation (“score”) is also given. The corresponding feature importances are reported in panels e–h, respectively. Abbreviations for the features are explained in the text. The samples are produced by spin coating in the fully robotic setup AMANDA Line1. The predictors mean: A_rPM6.o: amount of PM6 ordered phase, C_Y6.o: Y6 exciton energy, A_rDA: donor:acceptor ratio of oscillator strengths, W_Y6.o (W_Y6.a): width of vibronic peaks of Y6 exciton in ordered (amorphous) phase, A_tot: total oscillator strength; C_PM6.o: PM6 exciton energy. Reproduced with permission.^[^ ^80^ ^]^ Copyright 2021, Elsevier Inc.

Turner and coworkers have shown that UV–vis absorption spectra carry performance relevant information linked to morphology.^[^ ^87^ ^]^ The method is based on the model of weak H aggregates developed by F. Spano.^[^ ^88^ ^]^ Du et. al. have generalized and automated this method such that morphology relevant features can be extracted from donor‐acceptor blends in an automatic high throughput workflow without human intervention.^[^ ^80^ ^]^ Using Gaussian Process Regression (GPR), they were able to predict the electrical performance parameters (*V_OC_*, *J*_SC_, FF) of OPV devices based on PM6:Y6 even before the top electrode was deposited, see **Figure** **9**. Especially *V*_OC_ was predicted with high accuracy, see Figure 9b.

In ref. [80] feature importance was defined as the total effect of a parameter on the target value within the range of the dataset. The observed feature importance plots (Figure 9e–h) point to the underlying physics. Many of the observed relationships are expected and can be reproduced for example, by drift‐diffusion simulations:

1. The amount of ordered phase in the donor, A_rPM6.o, has a positive effect on the FF because a higher extraction mobility reduces charge accumulation and thus nongeminate charge recombination.
2. The total absorption A_tot, that is, the active layer thickness, has a negative effect on the FF. This is because larger thicknesses reduce the extraction field strength, causing a longer dwelling time of the charges, which leads to charge accumulation and nongeminate recombination.
3. C_Y6.o, the exciton energy of the Y6 ordered phase, has a positive effect on *V*_OC_ because it is related to the optical bandgap.

Apart from qualitative agreement with known physics models, a more quantitative look at the trends may help discovering new phenomena, or support alternative models currently in the discussion. An example is the negative influence of the PM6 exciton energy on *V*_oc_ in Figure 9f. This observation cannot be understood from simple physical models. From detailed balance considerations,^[^ ^89^ ^]^ the *V*_OC_ can be calculated as

(7) $$V_{OC}=E_{g,eff}\;+k_{B}T\;ln\left(\frac{{\left[np\right]}_{st}}{N_{eff}^{2}}\right)$$

where [*np*]_*st*_ is the product of the stationary densities of electrons and holes under 1 sun illumination and $N_{eff}^{2}$is the square of the effective density of states for electrons and holes. According to Equation (3), the donor bandgap, and thus the feature C_PM6_o – enters into Equation (7) via the donor HOMO energy. Assuming that the process conditions act on intermolecular interactions, a symmetric evolution of HOMO and LUMO levels would be expected according to the simple Hückel model. Increasing C_PM6_o would thus increase *E*_*g*,*eff*_, yielding a positive correlation of V_oc_ and C_PM6_o, in contrast to Figure 9f. We, therefore, need to look for physical models shifting HOMO and LUMO in the same direction, that is, abandon electronic to the favor of electrostatic effects, analogously to the results shown in Figure 4 by ref. [80] This notion does however not constitute a proof of the presence of electrostatic effects in the present dataset: first, we have considered only the variation of *E*_*g*,*eff*_ but ignored a possible variation of [*np*]_*st*_ in Equation (7). Second, an explicit quantum mechanical calculation of frontier orbital levels may expose deviations from the Hückel model. A suggested workflow to high throughput knowledge generation is thus to match candidate models to observed trends, in order to obtain a first hint for the most probable mechanism that can then be confirmed by the appropriate choice of supporting experiments. Acceleration comes from a more targeted approach to detailed experimentation driven by the results of high throughput studies.

High throughput knowledge generation relies on the input data and the resulting predictive models being representative for ground truth behavior, which presents an experimental challenge. Generally, regression analyses such as GPR require the data to be independent and isotropically distributed (i.i.d). In experimental design, this is often impossible: although we can design an experiment such that the process conditions are i.i.d, the resulting features (used to build predictive models) will have cross‐correlations and will often be sparse under a limited set of process parameters, that is, they evolve along a line rather than exhaustively sampling the available hypervolume. This notion does not entirely discourage a GPR analysis, but it restricts predictive capacity to a smaller hypervolume in which data are approximately i.i.d. Currently, these restrictions require careful manual data curation and validation; however efforts are ongoing to integrate data preprocessing and model building from multiple available evidences into a set of self‐learning agents, a so‐called Digital Twin for PV materials, for which a possible layout has recently been proposed.^[^ ^57^ ^]^

Du et al. also demonstrated that morphological stability can be predicted from optical features. It is well‐known that D:A blends involving the high‐performance NFA Y6 suffer from an initial burn‐in of the FF, which is ascribed to molecular rearrangements.^[^ ^33^ ^]^ Du et al. have shown that there is a significant correlation between morphology and the rate of burn‐in loss. Under photooxidative conditions, strong correlations between donor morphology and stability against photooxidation have been found.^[^ ^90^ ^]^

**Figure 10:**
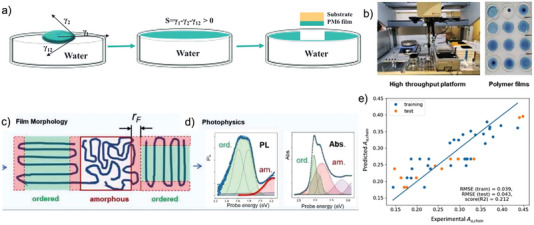
a) Principle of the Spreading Transfer Printing method. b) Experimental realisation in a high‐throughput platform and resulting samples (PM6 films). c) Distinction of extended amorphous regions with blue‐shifted PL and localized amorphous regions allowing Förster transfer into the ordered regions. d) Assignment of bands in the PL (left) and UV–vis spectra (right) to the different domains. The ratio of localized amorphous regions and ordered regions (A_a, chain_) should scale with the length of the ordered polymer chains. e) Gaussian Process Regression (GPR) to predict A_a,chain_ from the process conditions. Reproduced with permission.^[^ ^91^ ^]^ Copyright 2021, Wiley‐VCH.

In Figure 9e,g,h, the importance of ordering in the PM6 phase (predictor A_rPM6.o) becomes clear because it increases both FF and *J*_SC_. Therefore, a dedicated study on morphology control of PM6 films was performed. Wang et al.^[^ ^91^ ^]^ have deployed a novel method called Spreading Transfer Printing (STP) that relies on the spontaneous spreading of a polymer solution on a water surface, followed by film formation by solvent extraction toward the water and the gas phase.^[^ ^92^, ^93^, ^94^ ^]^ The floating film can then be transferred to a substrate that may already contain other layers, like, an acceptor layer. Using an automated HT platform, Wang et al varied process conditions (solvent mixtures, concentration, and temperature) and then used UV–vis and PL spectra to determine order parameters in the PM6 films, see **Figure** **10**a,b. By combining optical features from UV–vis and photoluminescence (PL) spectroscopy, see Figure 10d, two different amorphous phases were identified: extended amorphous phases, identified by blue‐shifted PL spectra due to suppression of Förster transfer into the lower energetic ordered phases, and localized amorphous phases so close to the ordered phase that complete Förster transfer into the ordered regions takes place suppressing the blue‐shifted PL.^[^ ^87^, ^95^ ^]^ The latter regions were associated with the folding region of the PM6 chains; their spectral weight, relative to the ordered regions, should therefore be a measure of the extension of the ordered regions, see Figure 10c. A GPR was performed (Figure 10e) to predict the relative length of the ordered chains from the process parameters, representing the different solvents by their physical properties such as the boiling point (a critical quantity for the drying time of the spread solution on the water surface), surface tension (a critical quantity for optimal spreading on the water surface) and Hansen parameters, determining the strength of interaction of PM6 with the solvent but also the speed of dissolution of the solvent into the water phase during drying. Using a bootstrap method to determine the uncertainty of the surrogate objective functions (non‐linear, multidimensional trends) found by GPR, the solvent boiling point was found as the only significant correlation with the length of the ordered PM6 chains. Interestingly, the boiling point of the solvent mixtures and that of the volatile additives followed the same trend, which can be rationalized by the dwelling time of the solvents and additives in the freshly formed films.

**Figure 11:**
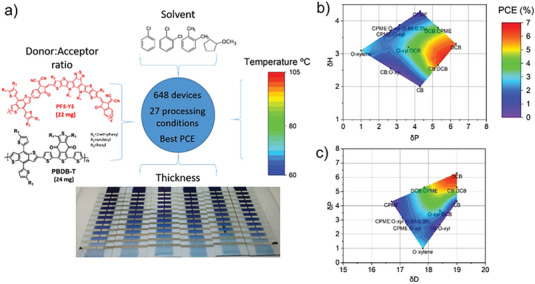
a) Schematic of the processing conditions for high‐throughput screening of polymer:polymer solar cells. b) and c) Performance landscape of polymer: polymer solar cells represented on the Hansen solubility space. Reproduced with permission.^[^ ^97^ ^]^ Copyright 2021, Wiley‐VCH.

The results shown in Figures 9 and 10 were obtained from samples produced by spin coating and STP, respectively, both being typical laboratory methods providing high versatility but cannot be upscaled for industrial production. Transfer of the technology from laboratory to production (“lab‐to‐fab”) therefore requires a transfer of the learned processing‐structure relationships to the industrial method. Harillo et al. have presented a HT study of variation of process conditions by blade coating, an upscalable method, producing >500 devices and varying 24 process conditions with the goal of maximizing PCE.^[^ ^96^, ^97^ ^]^ They optimized the polymer:polymer system PBDB‐T and PF5‐Y5 as donor and acceptor, respectively, see **Figure** **11**a. For all ‐polymer systems, microstructure optimization is particularly important.^[^ ^98^, ^99^ ^]^ Performing an ANOVA analysis, they found that the boiling point of the solvent mixtures was the most important processing parameter especially if the maximum, rather than the mean boiling point was assumed in solvent mixtures. This result was analogous to the one in ref. [91] where the maximum boiling point of solvent mixtures also was the strongest predictor. Moreover, Harillo et al identified clear trends of the PCE with the Hansen parameters of the solvent mixtures, see Figure 11b,c. The contour diagrams suggest an optimal combination of the Hansen parameters δP, δH, and δD for achieving high PCE values in this polymer‐polymer D:A system.

**Figure 12:**
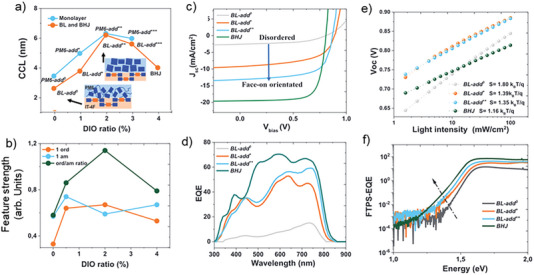
Morphology control of BL devices made of PM6:IT‐4F, where the IT‐4F layer is produced by spin coating and PM6 by STP. a) Crystal coherence length (CCL), as obtained from GIWAXS signals, as function of the amount of DIO additive; images in the inset show a pictorial representation of the molecular ordering as obtained from GIWAXS, b) integral UV–vis absorption of the first exciton transition of PM6 from the ordered region (orange), amorphous region (blue), and their ratio (black). c) Current–voltage curves, d) external quantum efficiencies, e) suns‐ *V_OC_* measurements. f) FTPS‐EQE spectra of BL devices of varying content of the DIO additive, compared to bulk heterojunction devices. Position and evolution of photocurrent generation by the interfacial CT state is indicated by a dashed arrow. Reproduced with permission.^[^ ^100^ ^]^ Copyright 2022, Wiley‐VCH.

Wang and co‐workers have systematically varied the donor morphology of BL devices of PM6:IT‐4F,^[^ ^100^ ^]^ as observed by optical spectroscopy and GIWAXS measurements, comparing the resulting Fourier Transform Photocurrent spectra (FTPS). The additive 1,8‐diiodooctane (DIO) was used to control the order in the PM6 phase, while the order of the IT‐4F phase was controlled by thermal annealing prior to transferring the PM6 layer and kept constant for both films. Treatment with DIO resulted in highly ordered and oriented PM6 phases, see inset in **Figure** **12**a. The ordering was assessed by the crystal coherence length (CCL), reaching a maximum of 6 nm for a concentration of 2% of DIO, see Figure 12a. The trend was corroborated by a maximum of the ratio of UV–vis absorption from ordered and amorphous regions at the same DIO concentration, see Figure 12b. This demonstrates that feature extraction from UV–vis spectra represents a powerful, cheap and fast proxy experiment to X‐Ray studies that are far more tedious and expensive and do not lend themselves to extraction of statistical significance.

Apart from the better ordering, GIWAXS patterns showed a preferential face‐on orientation of the PM6 chains with respect to the surface, whereas no preferential orientation in the PM6 phase was observed in the absence of DIO. The effect of the PM6 orientation on the electrical performance of the BL devices was drastic, see Figure 12c: highly ordered BL devices showed similar overall performance as the corresponding BHJ devices, clearly outperforming the BHJ in terms of *V*_OC_ but suffering from *J*_SC_ losses due to the incapacity of PM6 excitons to diffuse across a 50 nm thick layer to reach the D:A interface. The contribution of excitons generated in the donor and acceptor phases to the overall *J*_SC_ is identified by EQE spectra, see Figure 12d. If the PM6 phase was disordered, then EQE reached only ≈10% even for excitons created in the IT‐4F phase, where exciton quenching at the interface is not diffusion‐limited. In the case of disordered PM6 phases, it was found that a strongly negative bias was needed for efficient charge separation. Furthermore, suns‐ *V_OC_* measurements (Figure 12e) identified the prevalence of first order recombination for disordered PM6 phases, showing a lesser amount of bimolecular recombination at high intensities (hence the higher *V_OC_* values at 1 sun) but a higher amount of monomolecular recombination at low intensities, which may be related to the intrinsic lifetimes of CT states in equilibrium with LE states.

FTPS spectra (Figure 12f) showed a clear correlation of the spectral position and strength of the CT contribution with the ordering in the PM6 phase: face‐on oriented PM6 films showed stronger CT contributions at lower energy. As in BL devices, the number of CT states was fixed and given by the geometric area of the device (cross‐sectional TEM showing negligible roughness of the D:A interface), an increasing red‐shift of CT absorption is a clear sign of hybridization, fully in line with expectations from quantum chemistry. The results in BL devices thus showed that it is possible to fine tune the D:A interface by separately optimizing the morphology of both donor and acceptor phases.

## 6 Finding the Physical Root Cause for Device Performance and Degradation

**Figure 13:**
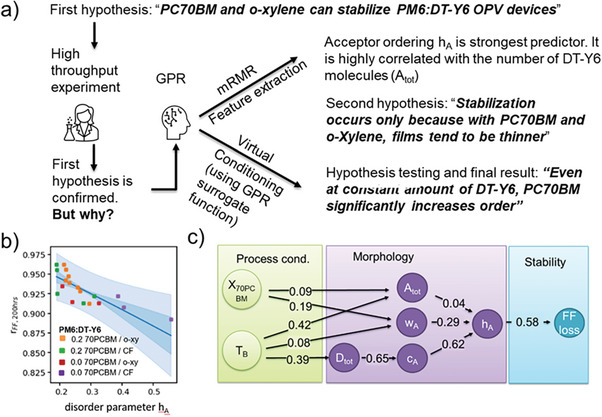
a) Integrated workflow for repeated hypothesis formulation and testing using a minimum number of experiments. b) Experimental data (symbols), showing the residual FF after 200 h (r_FF_, 200 h) as function of the strongest predictor h_A_. Different process conditions are color coded. Blue line: partial dependence of multidimensional surrogate function (as obtained by GPR) on h_A_; dark blue shaded region: uncertainty of the surrogate function (“trend error”), found by a bootstrapping method; light blue region: uncertainty of a single prediction (“y error”) c) Knowledge graph for the prediction of FF loss from morphological features, and of morphology from process conditions. The knowledge graph was obtained by recursively applying GPR from right to left, using minimum Redundancy Maximum Relevance (mRMR) feature extraction. The predictors are: X_70PCBM_: weight fraction of [70]PCBM, T_B_: boiling point of solvent, A_tot_: total absorption in exciton band of DT‐Y6, D_tot_: total absorption in exciton band of PM6, w_A_: spectral width of single vibronic of exciton band of DT‐Y6, c_A_: DT‐Y6 exciton energy, h_A_: Huang‐Rhys factor of single effective vibronic transition of DT‐Y6 exciton band. Adapted with permission.^[^ ^101^ ^]^ Copyright 2023, Wiley‐VCH.

In Figure 8, we indicated the presence of “hidden” process parameters, which are not under the control of the operators. If these parameters inadvertently change their values during the study, they will introduce uncertainty in the predictive capacity, reducing the strength of the observed correlations. Liu and coworkers have shown how this problem can be turned into a feature, allowing to distinguish direct from indirect causations for operational stability in ternary OPV devices made of the low driving force blend PM6: DT‐Y6,: [70]PCBM, see **Figure** **13**.^[^ ^101^ ^]^ Experimentally, it was found that admixture of [70]PCBM improves the operational lifetime of the devices. A further beneficial effect on the operational lifetime was found by replacing the standard solvent CF by o‐Xylene, a solvent with less stringent environmental regulations, an important consideration for production upscaling. However, across the whole dataset, a feature importance analysis identified the amount of DT‐Y6 molecules as one of the strong predictors for operational stability. Additionally, it was found that both, the admixture of the ternary component [70]PCBM and the use of o‐Xylene had a tendency to reduce the total amount of DT‐Y6 in the active layer. Therefore the question arose whether the direct cause of the improved operational stability by admixture of [70]PCBM or by using o‐Xylene, was really the lower amount of DT‐Y6. Thanks to the presence of one or more hidden parameters, the amount of DT‐Y6 molecules underwent fluctuations even when keeping the (known) process conditions constant. This makes it possible to “condition” the experiment on one of the parameters in question, for example, comparing the operational stability for the same amount of DT‐Y6 molecules but a different amount of [70]PCBM. Liu and co‐workers have shown that even in a limited dataset, this conditioning experiment can be performed virtually on the surrogate function whose uncertainty is encountered by running GPR with a bootstrap technique. Figure 13b shows the available data points (colored symbols) together with the surrogate function represented along the h_A_ axis assuming constant A_tot_, hence virtually conditioning the experiment to exclusively encounter the functional dependence of r_FF_ on h_A_. The light gray area shows the confidence interval of a single prediction for a given h_A_, whereas the dark blue area shows the confidence interval for the bootstrap uncertainty of the surrogate function itself. The confidence interval shows exclusively negative slope, which confirms the second hypothesis. Meanwhile, it was found that within the confidence intervals of the available dataset, the stabilization effect of o‐Xylene, as compared to CF, was only due to the higher film thickness realized in the latter solvent. Figure 13c shows a knowledge graph resulting from recursively applying a feature extraction technique called minimum Redundancy Maximum Relevance (mRMR) to identify the causal chain from process conditions (green) to structure (violet), and from structure to performance (blue). These results allow hypothesis testing with a minimum number of experiments, greatly accelerating the generation of knowledge.

Finally, we summarize recent progress in understanding and improving stability in D:A blends comprising NFA. An et al.^[^ ^102^ ^]^ highlight a critical type of morphology evolution under thermal stress, namely the clustering of the amorphous phase leading to accumulation of energetic traps, and show that it can be overcome by adjusting the blending ratio in a ternary blend TzBI‐dF:L8BO:Y6. Fan et al.^[^ ^103^ ^]^ developed a series of BTP derivatives featuring conjugated rather than aliphatic side groups, achieving efficiencies up to 18.3% in non‐halogenated solvents. These authors attribute the improved stability of the new compounds to steric hindrance. Conformational instability was also found to be a critical issue in some NFA compounds by Clarke et al.^[^ ^104^ ^]^ The stability of the desired bulk heterostructure was modeled by a molecular interaction–diffusion framework.^[^ ^105^ ^]^ The authors showed that the miscibility of the acceptor in the donor‐rich phase, described via the Flory‐Huggins parameter, is related to the diffusivity of the acceptor.^[^ ^104^ ^]^ If the miscibility is very low, then the diffusivity is also low. Surprisingly, this relationship leads to kinetic stabilization of those D:A systems whose miscibility is low, even though their optimized morphology (providing percolation in the donor phase) is far away from the thermodynamic minimum. In a broader study, Qin et al.^[^ ^106^ ^]^ compared stability trends in BTP derivatives. They used the glass transition temperature of the NFA and the elastic modulus of the donor polymer as proxy metrics to the molecular diffusion coefficients of NFA in the donor‐rich phase, to predict the kinetics of morphological evolution away from the percolation threshold due to over‐purification of the donor phase. The authors advocate for engineering new BTP derivatives with reduced diffusivity to avoid a burn‐in behaviour of electrical performance ascribed to vertical de‐mixing but highlight that reducing the diffusivity via side chain engineering is expected to negatively impact performance. Liang et al. thus chose a different approach to diffusivity regulation of BTP derivatives, namely developing oligomer acceptors, showing improved stability.^[^ ^107^ ^]^ In accelerated lifetime tests, well encapsulated and UV‐protected solar cells made of PCE‐10:BT‐CIC reached operational lifetimes over 30 years.^[^ ^108^ ^]^ A recent review discusses links between photoexcitation dynamics and stability, based on the resulting stationary density of unwanted degrading agents.^[^ ^109^ ^]^

## 7 Conclusion

We have presented a summary of the research efforts during the past years to understand and control charge separation in OPV at negligible driving force. The goal is to reduce voltage losses, which are still substantial despite the introduction of NFA, to approach those encountered in inorganic PV, where photoexcitation immediately creates charge separated states. There is an ongoing dispute about the exciton binding energy in NFA systems. The NFA transport gap, obtained by two different methods in Y6, is ≈0.3 eV, higher than the optical bandgap. This consolidated experimental finding contrasts with quantum mechanical calculations if considering solid state polarization, which results in near‐zero or even negative exciton binding energies. It also contrasts with the observation of intrinsic charge transfer in pure Y6, as well as in PM6:Y6 blends with high IQE values, when requiring to involve the assumption of entropic or disorder effects.

In contrast to the exciton binding energy, there is consensus about the nature of the driving forces for charge separation from the interfacial CT state in low driving force systems, with solid state effects (polarization enacted by electrostatics such as quadrupole moments) and hybridization being dominant players. In systems of low driving force, dynamic equilibria are in place between LE, CT and CS state. Given that only LE and CT states can deactivate to the ground state, and there is a much higher number of possible (bulk) LE than (interfacial) CT states, it is the LE lifetime that limits the achievable QFLS and thus defines the voltage losses. We have presented recent approaches to reduce vibronic and anharmonic coupling to vibrational modes in order to reduce nonradiative recombination rates following the energy gap Law.

Importantly, all three effects not only depend on molecular structures but also strongly on process conditions. This notion entails a huge search space not only for device optimization but also for knowledge generation, which encourages the use of high‐throughput methods assisted by artificial intelligence. We have presented various approaches to predict performance from process conditions, performance from microstructure, as well as microstructure from process conditions, obtained using different processing methods, namely floating film transfer, spin coating, and blade coating, which shows the broad applicability of the method. One difficulty of HT methods is that deep structural insight is hard to obtain because structure sensitive methods (for example by X‐ray scattering) typically do not lend themselves to HT workloads, also because data evaluation often involves expert interaction. In this review, we have presented two ways to tackle the issue: on one hand, the introduction of proxy experiments allows prediction of performance relevant microstructural details such as solid‐state ordering and energetic dispersion, from UV–vis or PL spectral shapes that can be obtained easily and automatically without human interaction. On the other hand, several authors have shown that critical system parameters such as the energies of the LE, CT, and CS states can be obtained indirectly, by concurrently matching several experimental evidence (spectra, dynamics, electrical performance) to a set of approximate solid‐state models (rate equations, drift‐diffusion models). Such approaches are highly in demand, because during the last years it has become clear that for a given molecule, these energies cannot be measured once and for all, but crucially depend on microstructural details and thus on the process conditions and the sample lifeline. These authors have demonstrated their method with small datasets. We expect that their method will unfold its full potential when combining it with HT workflows in a digital twin paradigm.

## Conflict of Interest

The authors declare no conflict of interest.

## Supporting information

Supporting Information
