# Supplementary material for: Maximizing Performance and Stability of Organic Solar Cells at Low Driving Force for Charge Separation
Source: Adv Sci (Weinh). 2023 Dec 1;11(6):2305948. doi: 10.1002/advs.202305948 (PMC10853714; doi:10.1002/advs.202305948)
Supplement: Supplementary file 1 — Supporting Information [file ADVS-11-2305948-s001.pdf]

ADVANCED SCIENCE
Open Access

Supporting Information

for *Adv. Sci.*, DOI 10.1002/advs.202305948

Maximizing Performance and Stability of Organic Solar Cells at Low Driving Force for Charge Separation

*Larry Lüer, Rong Wang, Chao Liu, Henry Dube, Thomas Heumüller, Jens Hauch and Christoph J. Brabec**

Maximizing Performance and Stability of Organic Solar Cells at Low Driving Forcefor Charge Separation

Larry Lüer,[a]Rong Wang,[a],[d]Chao Liu,[a], Henry Dube[b], Thomas Heumüller, [a]Jens Hauch, [c]Christoph J. Brabec[a],[c]*

[a] L. Lüer, R.Wang, C. Liu, C. J. Brabec
Institute of Materials for Electronics and Energy Technology (i-MEET), Friedrich-Alexander-Universität Erlangen-Nürnberg, Martensstrasse 7, 91058, Erlangen, Germany

[b] H. Dube, Department Chemistry & Pharmacy, Friedrich-Alexander-Universität Erlangen-Nürnberg, Nikolaus-Fiebiger-Straße 10, 91058, Erlangen, Germany

[c] J. Hauch, C. J. Brabec
Helmholtz-Institute Erlangen-Nürnberg (HI-ERN), Immerwahrstraße 2, 91058 Erlangen, Germany

[d] R. Wang. Erlangen Graduate School in Advanced Optical Technologies (SAOT), Paul-Gordan-Straße 6,91052 Erlangen, Germany

Correspondence to E-mail: christoph.brabec@fau.de

SUPPORTING INFORMATION

MOLECULAR STRUCTURES

Y6:2,2′-((2Z,2′Z)-((12,13-bis(2-ethylhexyl)-3,9-diundecyl-12,13-dihydro-[1,2,5]thiadiazolo[3,4-e]thieno[2,"3’’:4’,5′]thieno[2′,3′:4,5]pyrrolo[3,2-g]thieno[2′,3′:4,5]thieno[3,2-b]indole-2,10-diyl)bis(methanylylidene))bis(5,6-difluoro-3-oxo-2,3-dihydro-1H-indene-2,1-diylidene))dimalononitrile)

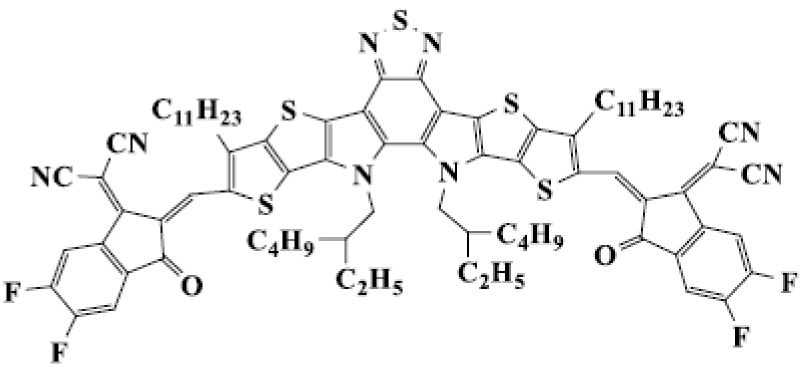

PM6: Poly[(2,6-(4,8-bis(5-(2-ethylhexyl-3-fluoro)thiophen-2-yl)-benzo[1,2-b:4,5-b'] dithio-phene))-alt-(5,5-(1',3'-di-2-thienyl-5',7'-bis(2-ethylhexyl)benzo[1',2'-c:4',5'-c']dithiophene-4,8-dione)]
